# Variable spin-charge conversion across metal-insulator transition

**DOI:** 10.1038/s41467-020-14388-9

**Published:** 2020-01-24

**Authors:** Taqiyyah S. Safi, Pengxiang Zhang, Yabin Fan, Zhongxun Guo, Jiahao Han, Ethan R. Rosenberg, Caroline Ross, Yaraslov Tserkovnyak, Luqiao Liu

**Affiliations:** 1 Department of Electrical Engineering and Computer Science, Massachusetts Institute of Technology, Cambridge, MA 02139 USA; 2 Department of Materials Science and Engineering, Massachusetts Institute of Technology, Cambridge, MA 02139 USA; 3 Department of Physics and Astronomy, University of California, Los Angeles, CA 90095 USA

**Keywords:** Magnetic properties and materials, Spintronics, Electronic properties and materials, Phase transitions and critical phenomena

## Abstract

The charge-to-spin conversion efficiency is a crucial parameter in determining the performance of many useful spintronic materials. Usually, this conversion efficiency is predetermined by the intrinsic nature of solid-state materials, which cannot be easily modified without invoking chemical or structural changes in the underlying system. Here we report on successful modulation of charge-spin conversion efficiency via the metal-insulator transition in a quintessential strongly correlated electron compound vanadium dioxide (VO_2_). By employing ferromagnetic resonance driven spin pumping and the inverse spin Hall effect measurement, we find a dramatic change in the spin pumping signal (decrease by > 80%) and charge-spin conversion efficiency (increase by five times) upon insulator to metal transition. The abrupt change in the structural and electrical properties of this material therefore provides useful insights on the spin related physics in a strongly correlated material undergoing a phase transition.

## Abstract (web summary)

The interconversion of spin and charge is fundamental to the operation of spintronic devices. Here the authors demonstrate spin-to-charge conversion in the correlated material vanadium dioxide, and show that the efficiency changes dramatically across the metal-insulator transition.

## Introduction

The functionality of spintronic devices is determined by three key processes: spin injection, spin manipulation, and spin detection. Since the pioneering spin injection experiment of Johnson and Silsbee in 1987, the focus has been to inject pure spin currents without any transfer of charge current in hopes of realizing low energy cost, high efficiency electronics^1^. Ferromagnetic resonance driven spin pumping is one of the most versatile and powerful tools to study the generation and detection of pure spin currents. Since its inception, people have utilized this technique to characterize the spin-orbit interaction induced charge—spin conversions in various materials and heterostructures, including paramagnetic, ferromagnetic and antiferromagnetic metals, semiconductors, and superconductors^2–9^. Recently, using materials with magnetic phase transitions as spin sinks, it has been demonstrated that the spin pumping efficiency undergoes a dramatic change across the transition point. This change was attributed to the change in magnetic ordering that modulates the spin mixing conductance at the ferromagnet/spin sink interface^10–14^. To date, most experiments related to spin-charge conversions are performed in materials with fixed structural and electrical properties. Having the additional ability to modulate the spin-charge conversion by varying these properties can lead to new functionalities for spintronic materials.

Vanadium dioxide (VO_2_) is a transition metal oxide with a unique property of a dramatic structural phase transition under external stimuli near room temperature. When the temperature rises above ~340 K, VO_2_ transitions from a monoclinic insulating phase into a rutile metallic phase accompanied with large and abrupt change in the electrical, optical, thermal, and magnetic properties^15–17^. Nowadays extensive research effort is geared at utilizing these properties for applications such as Mott transition field effect transistors, coupled relaxation oscillators for non-Boolean computing, etc^18–21^. Exploring the spin-charge conversion phenomena in this material can therefore open up potential applications as an active spintronic material. Moreover, this material provides a viable platform to study the fundamental spin related physics under phase transitions. The resistivity of VO_2_ varies by several orders of magnitude over a narrow temperature range across transition allowing an assessment of spin-charge conversion efficiency variation over a wide resistivity range. In addition, the structural change in VO_2_ (Peierls transition) is accompanied with an electron correlation transition (Mott transition), allowing us to probe the charge-spin conversion in a strongly correlated system^16^.

Here, through ferromagnetic resonance driven spin pumping experiments, we demonstrate variable spin-charge conversion (increase by five times) across the insulator-metal transition of VO_2._

## Results

### Sample structure and characterization

**Fig. 1:**
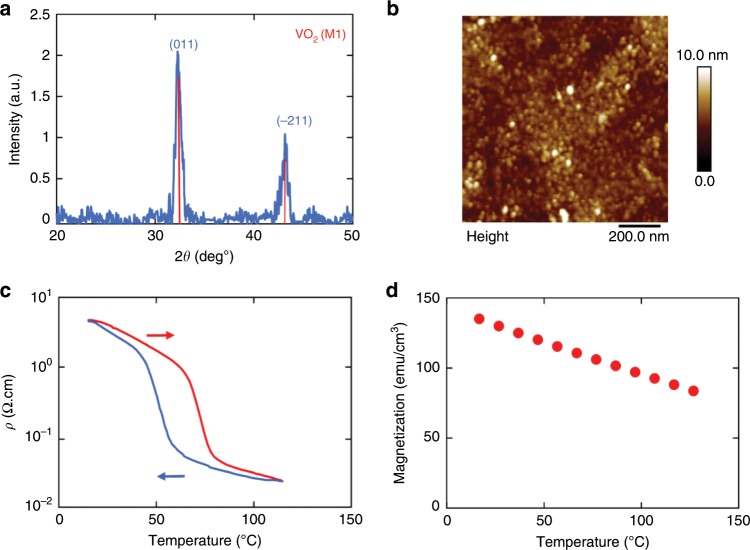
Structural, electrical, and magnetic properties of VO2 (68 nm)/YIG bilayer. **a** ***θ***−2*θ* X-ray diffraction scan (Cobalt source). **b** AFM image of VO_2_ (68 nm) surface. **c** Resistivity vs. temperature showing a metal-insulator transition between 62 and 77 °C. **d** Temperature dependence of the magnetization in VO_2_ (68 nm)/YIG(3 μm) at 1 kOe as measured from SQUID.

To study the spin-charge conversion properties of VO_2_, we started with YIG films grown on gadolinium gallium garnet (GGG) substrate with ultralow magnetic damping. Two different types of YIG films deposited with liquid phase epitaxy (~3 um thick) and radio frequency (RF) sputtering (100 nm thick) were employed in our experiment. Single phase, polycrystalline VO_2_ thin films (15–70 nm) were deposited on the YIG/GGG stack subsequently with RF sputtering (see methods). Figure 1a shows the X-ray diffraction of the sample, from which clear monoclinic phase peaks can be identified. We use atomic force microscopy to further examine the surface morphology, a smooth surface with an RMS roughness of 0.65 nm is obtained from the annealed samples (Fig. 1b). To characterize the metal-insulator transition in our VO_2_/YIG samples, we measure the resistivity, *ρ*, of the samples as a function of temperature *T*. Figure 1c illustrates *ρ(T)* result from a typical sample where a sharp decrease in resistivity (10^1^–10^−2^ Ω.cm) is observed at the transition temperature (*T*_t_) of *~*60 °C. The hysteresis in the *ρ* vs *T* curves from the subsequent heating and cooling scans is typical for the metal-insulator transition of VO_2_, reflecting the martensitic nature of the phase transition^22^. Magnetic measurements on YIG films were performed using SQUID magnetometer and were found to be unaltered pre- and post-deposition of VO_2_. Figure 1d shows the saturation magnetization (*M*_s_) of a 3 μm YIG film as a function of temperature after the deposition and annealing of VO_2_. The *M*_s_ value of ~140 emu/cc at 300 K is in good agreement with the values reported for single crystal YIG; furthermore, *M*_s_ changes by less than 30% across the temperature range of our experiment.

### Measurement setup

**Fig. 2:**
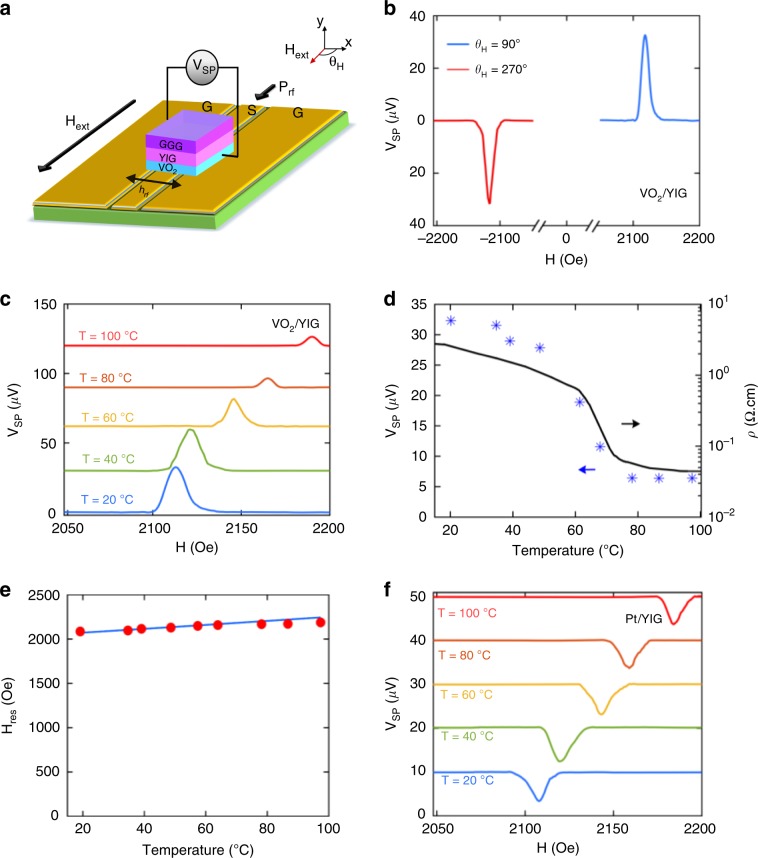
Schematic illustration of the spin pumping measurement and results. **a** Schematic of experimental setup for ISHE voltage measurements. **b** *V*_SP_ vs*. H*_ext_ spectra of VO_2_(68 nm)/YIG (3 μm) bilayer at *θ*_H_ = 90° (blue) and 270° (red) at *T* = 20 °C. **c** V_SP_ spectra of VO_2_ (68 nm)/YIG(3 μm) across the transition; the plots for different temperatures are shifted upwards by 30 *μ*V for clarity. Temperature dependence of **d** *V*_SP_ (stars) and resistivity (line) of theVO_2_(68 nm)/YIG(3 μm) sample (**e**) Resonant field from experiment (red) and calculated from Kittel’s formula (blue) at 8 GHz. (f) *V*_SP_ vs. *H*_ext_ for Pt(6 nm)/YIG bilayers sample at different temperatures; plots for different temperatures are shifted by 10 *μ*V for clarity.

The schematic of the setup used for the spin pumping measurement is illustrated in Fig. 2a. The samples are attached to a coplanar waveguide (CPW) with the VO_2_/YIG bilayer film facing down (see methods). At resonance, the spin current *J*_s_ from the magnetic resonance of YIG layer is injected along the *y*-axis into VO_2_ and is converted into a transverse spin pumping voltage (*V*_SP_) therein. Figure 2b shows the spin pumping spectra of a VO_2_(68 nm)/YIG(3 μm) bilayer at *θ*_H_ = 90° and 270° (in-plane fields) at *T* *=* 20 °C. As is expected, the inverse spin Hall effect (ISHE) voltage (*V*_SP_) changes sign upon the reversal of external magnetic field, *H*_ext_, due to the flipping of the injected spin orientation. Note that as YIG is an insulating ferromagnet there is no contribution from anisotropic magnetoresistance (AMR) and anomalous Hall effect (AHE) and the measured voltages are exclusively from the ISHE in VO_2_ layer. We also performed control experiments and ruled out the existence of other thermal contributions (see Supplementary Note 1).

### Observation of variable spin-charge conversion in VO_2_

**Fig. 3:**
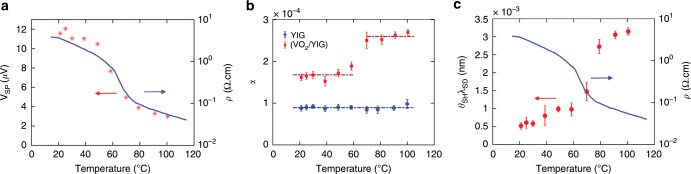
Variation of V_SP_ and charge-spin conversion across transition. Temperature dependence of **a** spin pumping voltage and resistivity of a VO_2_ (68 nm)/YIG (100 nm) sample, **b** Gilbert damping coefficient of bare YIG (blue) and VO_2_ (68 nm)/YIG (100 nm) (red) (the dash-dotted lines are drawn to guide the eye), and **c** variation of ***θ***_SH_*λ*_SD_ of VO_2_ (68 nm)/YIG (100 nm) sample across the metal-insulator transition. Error bars in this figure reflect uncertainties (standard error) in linear fitting of the damping coefficient.

To investigate the variation of spin pumping signal across metal-insulator transition of VO_2_, we vary the temperature of the sample with a ceramic heater, which is controlled by a PID controller with the temperature sensor mounted on the film surface. Figure 2c shows typical *V*_SP_ vs *H*_ext_ curves for a VO_2_(68 nm)/YIG(3 μm) sample between *T* = 20 °C and 100 °C at a microwave frequency of 8 GHz. The peak values of *V*_*SP*_ at different temperatures are summarized in Fig. 2d, where a large reduction of ~80% is observed throughout the temperature range. By comparing the *V*_SP_ variation with the resistivity change, we can clearly see that the dramatic decrease in *V*_SP_ coincides with the transition of VO_2_ from insulator to metal. In the transition region, *V*_SP_ lies between the extreme values of the metallic and insulating phases, reflecting the mixed state nature of this multi-domain regime. Besides the VO_2_/YIG(3 μm) sample, a similar transition behavior in spin pumping signal was also observed in samples with thinner YIG film (see Fig. 3a and Supplementary Note 2 for results on a VO_2_ (68 nm)/YIG(100 nm) sample), indicating that the observed phenomena does not rely on specific YIG film qualities. In addition to the change in the magnitude of *V*_SP_, we also observe a shift in the peak position of spin pumping signal as temperature varies, which can be explained by the reduction of *M*_s_ at higher temperatures. According to Kittel’s formula $\omega = \gamma \sqrt {H_{{\mathrm{res}}}\left( {H_{{\mathrm{res}}} + 4\pi M_{\mathrm{s}}} \right)}$^23^, for a fixed driving frequency, a decrease in *M*_s_ necessitates a higher external field to satisfy the resonance condition. The resonance field *H*_res_ as a function of temperature is plotted in Fig. 2e, which is in good agreement with the calculated curve using *M*_s_(*T*) data from Fig. 1d. The change in spin pumping voltage accompanying the phase transition does not depend on the driving frequencies of the microwave field, and is also consistent across VO_2_ films with different thicknesses (Supplementary Note 3 and 4). To exclude any systematic artifact, we also carry out parallel experiments on control samples of Pt/YIG films. As is evident from Fig. 2f, the magnitude of *V*_SP_ varies negligibly with increasing temperature in Pt/YIG sample, in sharp contrast to the results from VO_2_ samples (Supplementary Note 5). Moreover, we note that *V*_SP_ exhibits opposite signs for Pt and VO_2_ samples under the same measurement configuration, indicating that VO_2_ has a negative spin Hall angle, the same as pure metallic vanadium^9,24^.

The large change in the spin pumping voltage with temperature variation provides the first evidence of phase-dependent spin-charge conversions in this material, where the sample temperature is used as a tuning knob. We note that recently electric-field, irradiation or strain mediated phase transitions in VO_2_ has been shown in literature, where the higher switching speed (nanoseconds with voltage gating and sub-picoseconds with laser irradiation) and lower power consumption can help in realizing practical devices that are easier to modulate^25–30^. Recently, gate tunable spin-charge conversions in two-dimensional electron gas and ultrathin platinum have also been shown^31,32^. The discovery of a metal-insulator transition induced change of conversion efficiency in our experiment provides alternative mechanisms and material candidates for realizing this important goal.

The variable spin pumping signal across the metal-insulator transition requires a deeper understanding of the underlying mechanism that causes the aforementioned observations. In general, the spin pumping voltage depends on several fundamental material parameters such as resistivity, spin mixing conductance, spin Hall angle, etc. For example, an increase in the spin mixing conductance will enhance the injected spin current and raise the detected signal, while a decrease of resistivity across the transition can directly lower down the spin pumping voltage. In order to disentangle these factors and understand the observed variation, we start with the spin transport model in spin sink layer (VO_2_) under continuous spin generations from spin pumping^33–35^. Assuming that the thickness of the spin sink layer is much larger than its spin diffusion length, the measured inverse spin Hall voltage is given by^9,33^,

1 $$V_{{\mathrm{ISHE}}} = - \frac{{{\mathrm{e}}\;\theta _{{\mathrm{SH}}}}}{{\sigma _{\mathrm{s}}t_{\mathrm{s}}}}\lambda _{{\mathrm{SD}}}g_{{\mathrm{eff}}}^{ \uparrow \downarrow }f\;L\;P\;{\mathrm{sin}}^2\theta _{\mathrm{m}}$$

Here, *e* is the electron charge, *σ*_S_, *t*_S_ and ***λ***_SD_ are the conductivity, thickness and spin diffusion length of spin sink, ***g***_eff_^↑↓^ is the effective spin mixing conductance, *L* is the sample length, *f* is the microwave frequency, and ***θ***_m_is the magnetization precession cone angle. At resonance, $\theta _{\mathrm{m}} = \frac{{2h_{{\mathrm{rf}}}}}{{{\mathrm{\Delta }}H}}$ where**Δ***H* is the linewidth and *h*_rf_ is the microwave magnetic field^33^. Using the calibrated rf magnetic field in our experiment and the FMR linewidth, we determine that *θ*_*m*_~0.008^*o*^. *P* is a correction factor accounting for the ellipticity of the magnetization precession, which is calculated to be *P* ≈ 1.09 ± 0.04 using the relationship given in ref. ^36^.

The spin mixing conductance, ***g***_eff_^↑↓^, describes the flow rate of spins pumped into the spin sink, which leads to the generation of *V*_ISHE_. As the spin mixing conductance originates microscopically from the exchange interaction of electrons across the ferromagnet/spin sink interface, a difference is expected between a metal-insulator interface and an insulator-insulator interface. Since spin pumping adds to the bulk intrinsic damping ***α***_YIG_ of the ferromagnet, we can experimentally quantify ***g***_eff_^↑↓^ by measuring the enhancement in magnetic damping, $\alpha _{{\mathrm{YIG}}/{\mathrm{VO}}_2} - \alpha _{{\mathrm{YIG}}}$, which leads to $g_{{\mathrm{eff}}}^{ \uparrow \downarrow } = \frac{{4{\uppi}M_{\mathrm{s}}t_{\mathrm{F}}}}{{g\mu _{\mathrm{B}}}}(\alpha _{{\mathrm{YIG}}/{\mathrm{VO}}_2} - \alpha _{{\mathrm{YIG}}})$^33,36^, where *g*, *μ*_B,_ $\alpha _{{{\mathrm{VO}}_2}/{\mathrm{YIG}}}$, and *α*_YIG_ are the Landé factor, Bohr magneton, and Gilbert damping coefficient for VO_2_/YIG bilayer and bare YIG, respectively. We note that the equation above only holds for thin YIG samples whose thickness ***t***_F_ is smaller than the ferromagnet coherence length, which is on the order of a few hundred nanometers, as the dynamics of magnetic moment that are far away from the ferromagnet/spin sink interface do not contribute to the spin pumping^37^. In order to get an accurate determination of the value of ***g***_eff_^↑↓^, we extract the Gilbert damping coefficients ***α*** for thin sputtered YIG films. ***α*** is related to the ferromagnetic resonance linewidth *ΔH* at different resonance frequencies through the formula: ${\mathrm{\Delta }}H = {\mathrm{\Delta }}H_{{\mathrm{inh}}} + \frac{{4\pi \alpha f}}{\gamma }$^38^ (see Supplementary Note 6). The extracted damping coefficients for a bare YIG sample and a VO_2_ (68 nm)/YIG(100 nm) sample at different temperatures are summarized in Fig. 3b. It is noted that the damping coefficient of bare YIG remains largely unchanged within the temperature range, whereas the VO_2_/YIG sample exhibits a sudden increase when the temperature rises above *T*_t_, indicating an enhancement in spin pumping across the metal-insulator transition. In our experiment, the effective spin mixing conductance changes from (4.0 ± 0.20) × 10^17^*m*^−2 ^to (8.5 ± 0.20) × 10^17^*m*^−2^ as the temperature increases. The increase in ***g***_eff_^↑↓^for higher temperature is consistent with the picture of a more transparent interface in the metallic regime due to the larger density of states of conduction electrons. Here, we recognize that the standard drift-diffusion model may not accurately describe spin transport in a high resistivity material such as VO_2_, where the electron hopping time can be significantly longer than the spin decay time. However, if the same spin-orbit coupling mechanism dominates the spin relaxation leading to Gilbert damping enhancement and the spin pumping voltage *V*_ISHE_, they can still be correlated through a single phenomenological parameter ***g***_eff_^↑↓^ (see Supplementary Note 6).

The spin diffusion length, ***λ***_SD_, quantifies the decay of the spin accumulation into the spin sink and its value directly determines the active region for the spin to charge conversion. Experimentally ***λ***_SD_ is usually determined by measuring the spin pumping voltage in samples with different spin sink thicknesses. We note that studying the thickness dependence of spin pumping signal in VO_2_ remains a challenging task, as the phase transition temperature of a VO_2_ film is sensitively dependent on the film thickness^25,39^. Upon varying the thickness, we observe a simultaneous change in the film resistivity as well as the transition amplitude and sharpness, which indicates a change in the intrinsic properties of the films. The decrease in transition amplitude and sharpness is more pronounced for film thicknesses below 40 nm, which is the thickness range where the spin diffusion length may exert major influence (see Supplementary Note 3). Therefore, it is not feasible to directly quantify ***λ***_SD_ by varying the film thickness. Instead, to quantify the spin to charge conversion in this material we utilize the parameter of ***θ***_SH_*λ*_SD_,which was introduced in earlier studies for comparison of systems with 2D and 3D transport^31^. We note that this product of ***θ***_SH_ and ***λ***_SD_ represents the figure of merit of spin pumping experiment, as it directly reflects the efficiency of generating inverse spin Hall effect voltage for a given injected spin current. As is shown in Fig. 3b, ***θ***_SH_*λ*_SD_ increases by more than five times across the measured temperature range, indicating a large enhancement in the spin to charge conversion efficiency. A similar treatment of our control sample of Pt/YIG yields an ***θ***_SH_*λ*_SD_ value of 0.45 nm and the spin Hall angle of Pt ***θ***_SH_ = 0.065 ± 0.005 (***λ***_SD_ ≈ 7*nm*), which are comparable to the reported values^40^. In contrast to the abrupt change across the MIT transition for VO_2_, the spin Hall angle exhibits no noticeable change over the temperature range for Pt (Supplementary Note 5).

The spin Hall effect has different scaling behaviors under different detailed mechanisms^41,42^. In the intrinsic regime, the spin Hall conductivity ***σ***_xy_^SH^ remains constant and spin Hall angle***θ***_SH_~*σ*_xy_^SH^/*σ*_xx_ increases as the longitudinal conductance ***σ***_xx_ decreases^43^. Therefore, one can enhance the charge-spin conversion efficiency by making more resistive materials via impurity doping or alloying in this regime^44–46^. In contrast, within our studied material, the spin to charge conversion, as is quantified by ***θ***_SH_*λ*_SD_, shows an opposite trend and it is higher for a larger ***σ***_xx_, indicating that VO_2_ resides in the electron hopping/dirty metal regime. Therefore, to further improve the conversion efficiency in VO_2_, films with higher quality and larger metallic phase conductivity will be helpful.

To conclude, we have experimentally demonstrated phase-dependent spin-charge conversions in VO_2._ We show that upon the metal-insulator transition, spin mixing conductance and spin-charge conversion efficiency exhibit an abrupt change. The large (approximately five times) change in the spin-charge conversion within the same material brings forth opportunities for engineering spintronic materials with variable functionalities. Even larger variations in the conversion efficiency are expected in optimized VO_2_ films with higher quality and sharper phase transitions.

## Methods

### Sample preparation

The samples used in this study are yttrium iron garnet (YIG)/ VO_2_ bilayer films. YIG films with thicknesses in the range of 2.5–3 μm were grown on gadolinium gallium garnet (GGG) substrate using liquid phase epitaxy. Thinner YIG samples with thicknesses ranging from 55 to 100 nm were grown using rf-sputtering from a YIG target. The samples were subsequently annealed in a furnace for two hours at 850 °C in 100% O_2_.VO_2_ thin films (15–70 nm) were sputtered on the YIG/GGG stack using radio frequency sputtering from a VO_2_ target. The advantage of using a VO_2_ target, as compared to reactive sputtering, is the stoichiometric stability of the deposited film which is determined mainly by the V–O stoichiometry in the sputtering target. The substrate (YIG/GGG stack) was kept at 300 °C during deposition. The films were sputtered at a working pressure of 5 mTorr (Ar gas) and subsequently annealed by rapid thermal treatment at 450 °C for 2 min in an O_2_ (4%) + N_2_ (96%) mixture.

### Resistivity measurements

Four-point resistivity measurements were carried out on unpatterned VO_2_ (4 × 4 mm^2^) films (Fig. 1c). The samples were placed on a temperature-controlled stage with a thermocouple attached to the top of sample surface. At each temperature, resistivity was extracted from I–V curves while maintaining the linearity of I–V curves to exclude joule heating.

### Ferromagnetic resonance (FMR) measurements

The measurement setup is depicted in Fig. 2a. For FMR measurements, the DC magnetic field was modulated with an AC field of 0.5 Oe at 199 Hz. The transmitted signal from the CPW was detected by a lock-in amplifier via an RF-diode detector. We observed the FMR spectrum of the sample by sweeping the external magnetic field. The data obtained were then fitted to a sum of symmetric and antisymmetric Lorentzian functions to extract the linewidth.

### ISHE measurements

The schematic of the setup used for spin pumping measurement is illustrated in Fig. 2a. The samples are attached to a coplanar waveguide (CPW) with the VO_2_/YIG bilayer film facing down. The CPW is further placed between electromagnet pole pieces that provide homogeneous DC external magnetic field (*H*_ext_) in sample plane (*xz*). The ISHE voltage is measured across the VO_2_ layer along the *x*-axis. For temperature dependent measurements, we mounted the CPW onto a ceramic heater. The sample temperature was monitored with a thermocouple attached to the film surface and carefully controlled with a PID temperature controller. At each setpoint the temperature was maintained within ±0.5 °C. We observed no significant variation in the transmission coefficient of the microwave signal through the CPW over the experimental temperature range of 20–120 °C, indicating negligible changes in the driving power of spin pumping. We excited the FMR by sweeping the external magnetic field while fixing the excitation microwave frequency (between 3 and 10 GHz), and measured the ISHE voltage across the sample with a DC voltmeter.

## Supplementary information

Supplementary Information

## Data Availability

The data that support the findings of this study are available from the corresponding author upon request.
