# Supplementary material for: Variable spin-charge conversion across metal-insulator transition
Source: Nat Commun. 2020 Jan 24;11:476. doi: 10.1038/s41467-020-14388-9 (PMC6981235; doi:10.1038/s41467-020-14388-9)
Supplement: Supplementary file 1 — Supplementary Information [file 41467_2020_14388_MOESM1_ESM.pdf]

Supplementary Information

Variable spin-charge conversion across metal-insulator transition

Safi et al.

Table of Contents

Supplementary Note 1. Control experiments to exclude heating effects

Supplementary Note 2. Spin pumping measurement for VO_2(68nm)/YIG(100nm)

Supplementary Note 3. Spin pumping results for various VO_2 film thicknesses

Supplementary Note 4. Spin pumping results for different driving frequencies

Supplementary Note 5. Control Sample Pt/YIG

Supplementary Note 6. Extraction of Gilbert Damping Coefficient

Supplementary Note 7. Carrier Density Across Transition

Supplementary Note 1. Control experiments to exclude heating effects

Using an insulating ferromagnet such as YIG has the advantage of exclusion of contributions from anisotropic magnetoresistance (AMR) and the anomalous Hall effect (AHE) to the ISHE voltage in the spin sink. However, there is still a possibility that the measured signal is altered by the existence of thermal effects.

In our experimental configuration as depicted in main text Fig.2(a), the in-plane magnetization is along z-axis. Any temperature gradient along y-axis, i.e., in the out of plane direction can lead to a possible anomalous Nernst effect voltage along x-axis given by $\Delta V_{\mathrm{ANE}} \propto \Delta T \cdot M$. This ANE contribution could be significant in magnetic metal samples such as permalloy. However, the use of ferromagnetic insulator, YIG, rules out such a contribution. The voltage arising from the ordinary Nernst effect, where the charge current is driven by a thermal gradient can also have the same symmetry as V_{SP}, as is suggested in recent literature [1]. To rule out contributions from the Nernst effect, we made an additional control sample by inserting a thin MgO film (3nm) between YIG and VO_2. This will suppress any spin transport from YIG to VO_2 and any remaining voltage will be due to V_{NE}. From Fig. 1, we see that under the same experimental conditions as in the main text, the ISHE voltage vanishes for the VO_2(68nm)/MgO(3nm)/YIG (100nm) sample. This rules out contributions from the ordinary Nernst effect stemming from VO_2 layer.

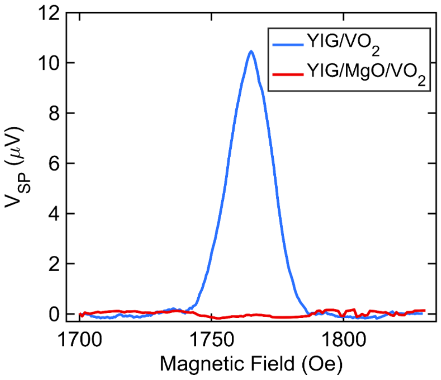

Figure 1. Comparison between spin pumping voltages of YIG/VO_2 samples with and without MgO insertion. The sample with MgO barrier shows no detectable spin pumping voltage.

Supplementary Note 2. Spin pumping measurement for VO_2(68nm)/YIG(100nm)

Fig. 2 shows the spin pumping signal measured for the thinner YIG sample discussed in main text. We see that the spin pumping signal amplitude drops concurrently with the metal insulator transition. This is qualitatively consistent with the main text results for a VO_2 (68 nm)/YIG (3 μm) sample, considering the thinner YIG films used here.

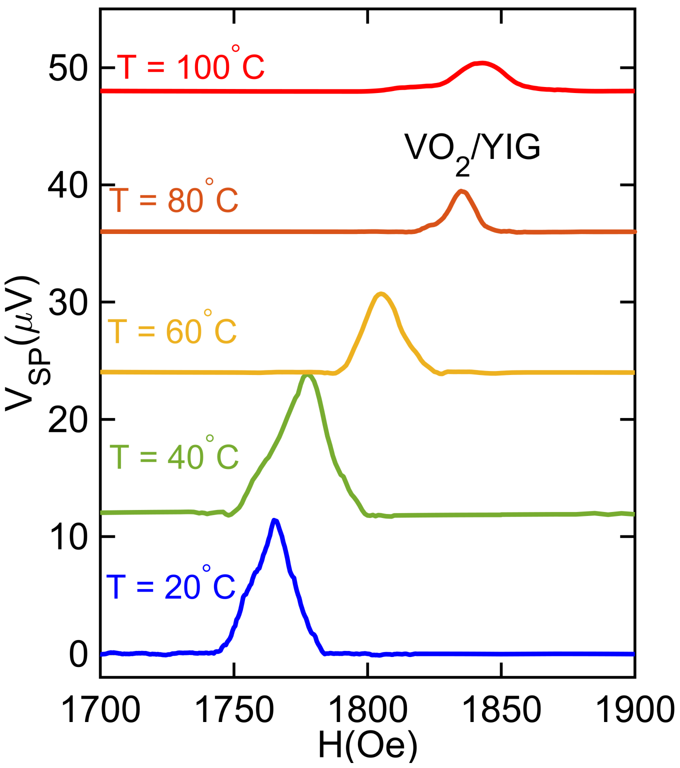

Figure 2. Temperature dependence of V_{SP} in VO_2(68 nm)/YIG(100 nm) bilayers. Plots for different temperatures are shifted for clarity.

Supplementary Note 3. Spin pumping results for various VO_2 film thicknesses

VO_2 films with varying thicknesses were grown on YIG substrate. Following the discussion in main text, it's evident from Fig. 3 that the transition temperature and sample resistivity is different for the films with varying thickness. First, we note that the YIG substrate is not ideal for the growth of VO_2 due to the unmatched lattice structures. We see that thinner VO_2 films have a less sharp transition and a smaller transition amplitude. This behavior is expected in poor quality VO_2 films and has been studied extensively: as the crystal quality degrades (smaller grain size, more defects), the hysteresis width increases while transition amplitude decreases [2-3]. This is attributed to increased scattering from smaller grain size as well as discontinuity (defects) in thin films.

Fig. 3 shows spin pumping data for additional samples of VO_2 with different film thicknesses deposited on YIG than the main text results. Despite the reduced VO_2 quality in thinner films, we find that the spin pumping voltage always decreased concurrently with the phase transition, indicating that it's a universal behavior for metal-insulating transition in vanadium dioxide material. Following the main text, we extracted the $\theta_{\mathrm{SH}}\lambda_{\mathrm{SD}}$ parameter for thinner samples and see that qualitatively the trend is the same as the thicker samples. From Fig. 3, we see that as the transition amplitude decreases and the transition width increases in thinner samples, the variation in $\theta_{\mathrm{SH}}\lambda_{\mathrm{SD}}$ also becomes less sharp and its magnitude change across transition decreases as well. From this we infer that higher quality VO_2 films with a higher resistivity change and smaller transition width will give a larger, more abrupt contrast in spin-charge conversion across the insulator to metal transition. Furthermore, in the standard approach, λ_{SD} is extracted from the thickness dependence of inverse spin Hall voltage through: $\frac{V_{\mathrm{ISHE}}}{Rw} \propto \lambda_{\mathrm{SD}} \tanh\left(\frac{t_s}{2\lambda_{\mathrm{SD}}}\right)$. As the film quality is not consistent across the range of thicknesses, it is hard to get a reliable value for λ_{SD} using this method for our samples.

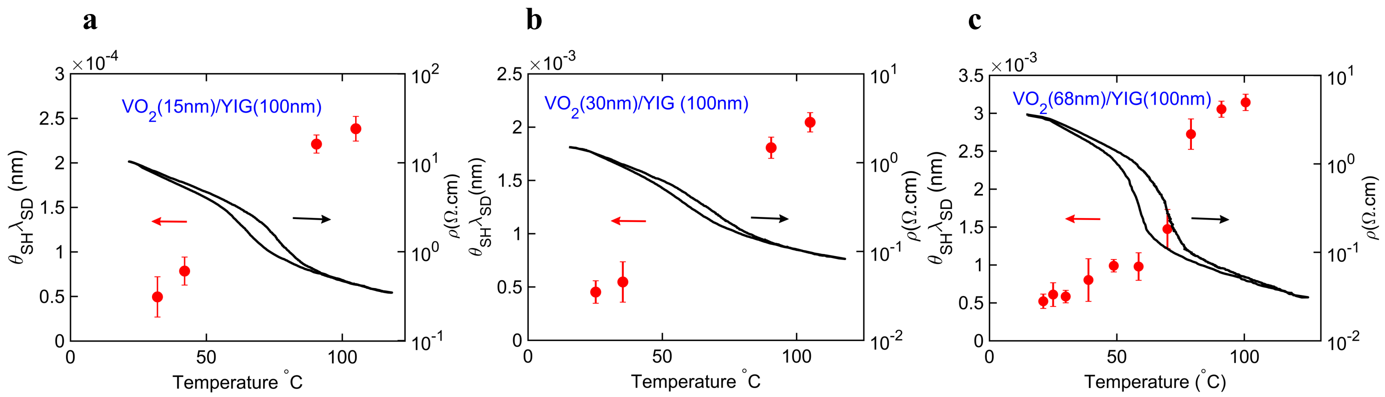

Figure 3. Spin charge conversion in VO_2 films with different thicknesses. $\theta_{SH}\lambda_{SD}$ and resistivity as a function of temperature for (a)VO_2 (15 nm)/YIG(100 nm) (b)VO_2 (30 nm)/YIG(100 nm) and (c)VO_2 (68 nm)/YIG(100 nm) across the metal-insulator transition. Error bars in the figures reflect uncertainties (standard error) in linear fitting of the damping coefficient.

Supplementary Note 4. Spin pumping results for different driving frequencies

The spin pumping voltage signal measured at different driving microwave frequencies is summarized in Figure 4a. It is clear that there is no frequency dependence. Furthermore, following the discussion in main text we calculate the $\theta_{SH}\lambda_{SD}$ product and the trend holds for different frequencies and the magnitude is comparable within the experimental uncertainty limits.

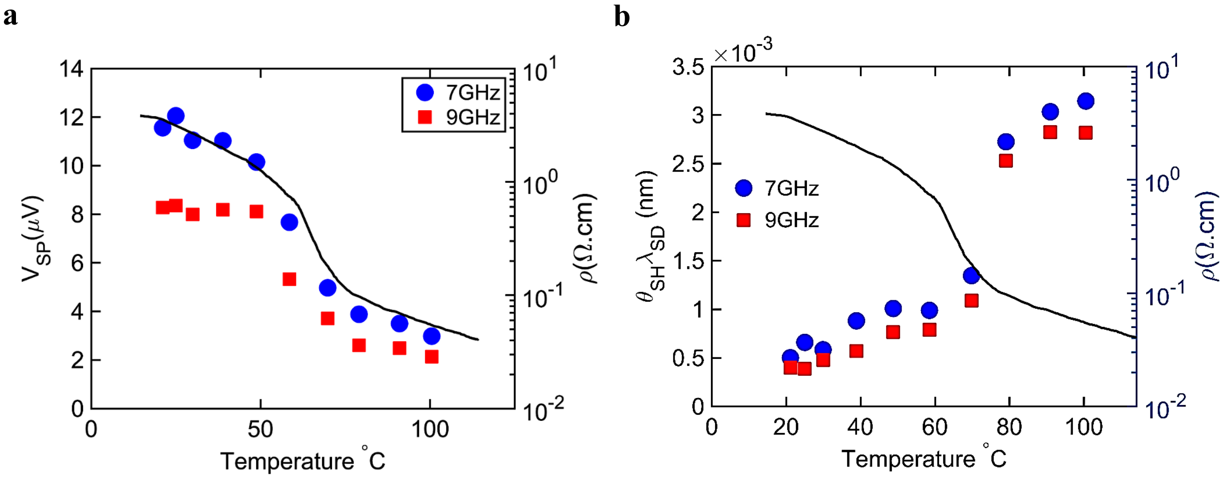

Figure 4. Spin pumping at different driving frequencies. (a) Temperature dependence of the spin pumping signal at 5 and 10 GHz for VO_2(68 nm)/YIG(100 nm). The temperature dependence of resistivity is plotted as a solid line as well. (b) The variation of $\theta_{SH}\lambda_{SD}$ and resistivity of VO_2 (68 nm)/YIG(100 nm) across the metal-insulator transition measured at different frequencies.

Supplementary Note 5. Control Sample Pt/YIG

The temperature dependence of spin pumping voltage from a Pt (6nm)/YIG sample is summarized in Fig. 5a. The spin pumping voltage has negligible change over the temperature range of our experiment. The spin Hall angle is calculated following the procedure in main text using a spin diffusion length of $\lambda_{\mathrm{SD}} = 7nm$ [4].

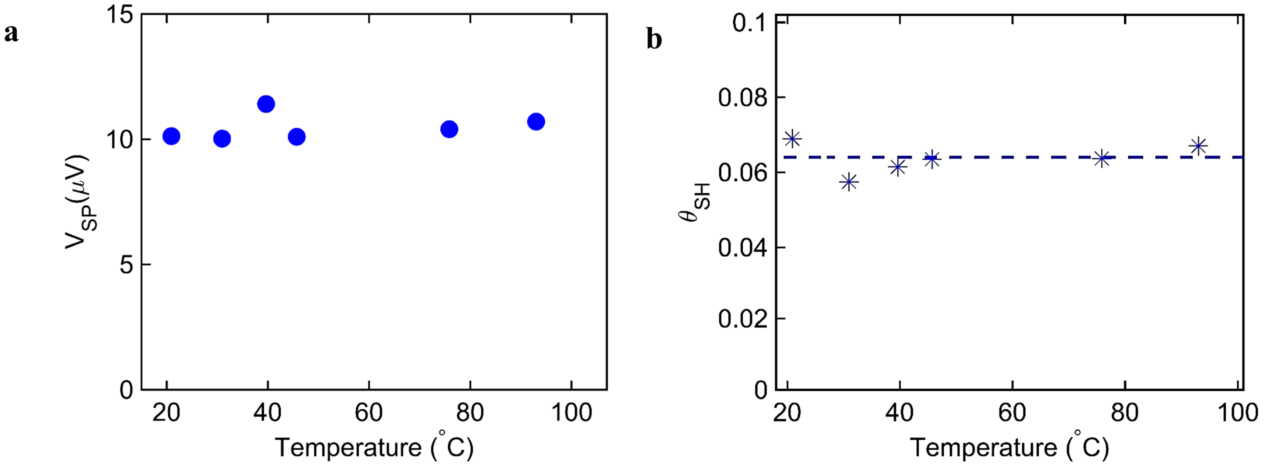

Figure 5. Temperature dependent measurements of Pt/YIG bilayers. (a) Temperature dependence of V_{SP} of a Pt(6 nm)/YIG control sample, and (b) calculated spin Hall angle. The dash-dotted line represents average value.

Supplementary Note 6. Extraction of Gilbert Damping Coefficient

Following the discussion in main text, the Gilbert damping coefficient at each temperature is extracted from the measured ferromagnetic resonance linewidth ΔH at different resonance frequencies. The least square fit to the formula $\Delta H = \Delta H_{\mathrm{inh}} + \frac{4\pi\alpha f}{\gamma}$ leads to the damping coefficient plotted in the main text [4-6]. The linewidth and linear fit for both VO_2/YIG bilayer and bare YIG at different temperatures is shown in Fig.6. In Fig. 6a, for the VO_2/YIG bilayer the fit slope is larger at 90 °C than 40 °C indicating an increase in the damping coefficient after the phase transition. In contrast, for the bare YIG sample we see no such trend. We also observe an increase in resonant linewidth with decreasing temperature; this is a trend that has been observed before and has been ascribed to the slowly relaxing impurity mechanism and surface-induced relaxation mechanisms [7-9].

In the standard spin diffusion model, the effective spin mixing conductance as determined in the main text is constituted as $\frac{1}{g_{\mathrm{eff}}^{\uparrow\downarrow}} = \frac{1}{g^{\uparrow\downarrow}} + \frac{1}{g_{\mathrm{s}}}$, where $g^{\uparrow\downarrow}$ is the bare interfacial spin-mixing conductance and g_{s} is the conductance of the normal-metal region of thickness λ_{SD} as described in reference [34] of main text. For a highly resistive material as studied in our case, the standard diffusive model may not apply as the electron hopping time can be potentially longer than the spin dephasing time. While decomposing $g_{\mathrm{eff}}^{\uparrow\downarrow}$, into the interfacial part and g_{s} part as in the standard model is not always granted, the damping enhancement and spin pumping voltage V_{ISHE}, can still be correlated through a single phenomenological parameter $g_{\mathrm{eff}}^{\uparrow\downarrow}$, given that the spin relaxation mechanism which leads to Gilbert damping enhancement and the one which induces spin Hall current originate from the same spin-orbit coupling physics. We note that the usage of this effective spin mixing conductance is also utilized in previous experiments which involve resistive materials such as semiconductor and organic films [10-15]. On the other hand, if there is any additional spin relaxation mechanism that does

not lead to spin Hall effect at the interface such as spin memory loss, the injected spin current will be smaller than the one calculated from damping enhancement, therefore, the spin/charge conversion efficiency determined in our experiment should be understood as the lower bound of real values.

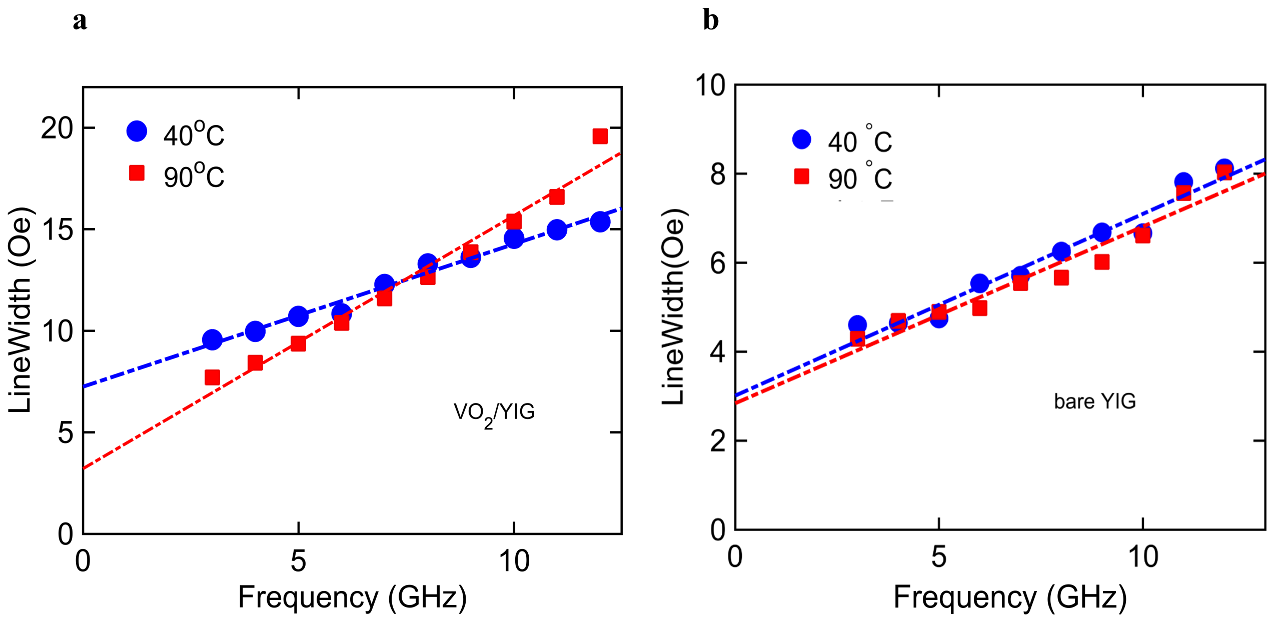

Figure 6. FMR linewidth versus frequency: The FMR linewidth versus frequency at pre- (blue circles) and post- transition temperatures (red squares) for (a) VO_2(68 nm)/YIG(100 nm) and (b) bare YIG(100 nm) samples.

Supplementary Note 7. Carrier Density Across Transition

We extracted the carrier density of our VO_2(68 nm)/YIG films from Hall measurements. We found that electrons are the major carriers on both sides of the transition and the carrier density changes by ~3 orders of magnitude across transition as shown in Fig. 7. This is in agreement with reported carrier type and density values in literature [16,17]. This change in carrier concentration directly contributes to the variation in the resistivity, which further influences the measured V_{ISHE} in our experiment. Besides the resistivity contribution, which is taken into account during our calculation of spin to charge conversion efficiency ($\theta_{SH}\lambda_{SD}$), the carrier concentration may influence the spin to charge conversion in additional ways. For example, the filling of additional carriers in the insulating state could change the Fermi level position, which will modify the Berry curvature contribution, in a way similar to Rashba effect and topological insulator system.

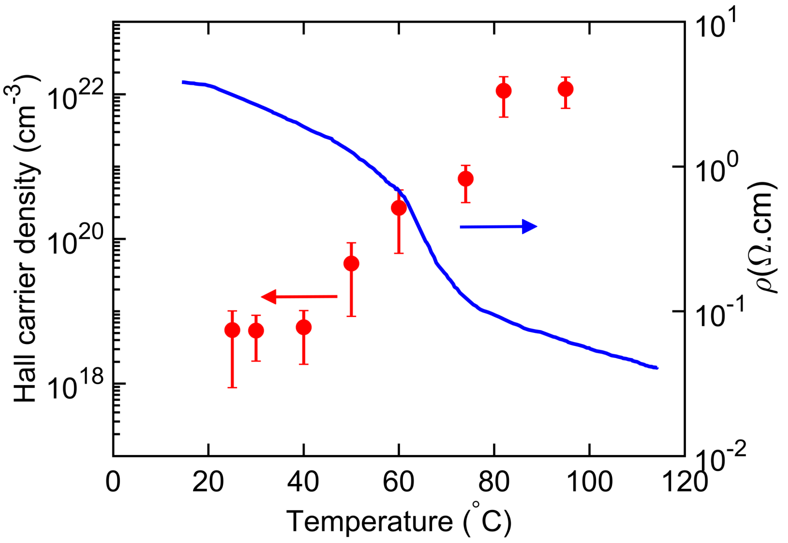

Figure 7. Carrier density across transition: The Hall carrier density as a function of temperature measured across the insulator to metal transition for VO_2(68 nm)/YIG(100 nm). Error bars in this figure reflect standard deviation of the experimental data.

Supplementary references

[1] Di Yue, Weiwei Lin, Jiajia Li, Xiaofeng Jin, and C. L. Chien. *Phys. Rev. Lett*. 121, 037201 (2018)

[2] D. Brassard et al,. *Appl. Phys. Lett.* 87, 051910 (2005)

[3]. V.S. Vikhnin et al,. *Phys. Lett*. A. 343(6), 446–453 (2005)

[4]. H. L. Wang, C. H. Du, Y. Pu, R. Adur, P. C. Hammel, and F. Y. Yang, *Phys. Rev. Lett.* 112, 197201 (2014).

[5] Y. Tserkovnyak, A. Brataas, and G. E. W. Bauer, *Phys. Rev. Lett.* 88, 117601(2002).

[6] S. S. Kalarickal, P. Krivosik, M. Wu, C. E. Patton, M. L. Schneider, P. Kabos, T. J. Silva, and J. P. Nibarger, *J. App. Phys.* 99, 093909 (2006).

[7] C.L. Jermain, S. V. Aradhya, N.D. Reynolds, R. A. Buhrman, J. T. Brangham, M. R. Page, P. C. Hammel, F. Y. Yang, D. C. Ralph, *Phys. Rev.* B, vol. 95 174411 (2017).

[8] E. G. Spencer, R. C. LeCraw, A. M. Clogston, *Phys. Rev. Lett.,* 3, pp.32-33 (1959).

[9] N. Beaulieu, N. Kervarec, N. Thiery, O. Klein, V. Naletov, et al, *IEEE Mag. Lett.* 9, pp.3706005 (2018)

[10] R. Ohshima et al. *Appl. Phys. Lett*. 110, 182402 (2017)

[11] J.B.S.Mendes, A. Aparecido-Ferreira*,* J. Holanda*,* A. Azevedo*,* S. M. Rezende. *Appl. Phys. Lett.* 112, 242407 (2018)

[12] A. Jain et al. *Phys. Rev. Lett*. 109, 106603 (2012)

[13] M. C. Wheeler et al. *Nat. Comm.* Vol 8, 926 (2017)

[14] D. Sun et al. *Nat. Mater.* Vol 15, pg 863–869 (2016)

[15]. J.C. Lee et al. *Appl. Phys. Lett*. 104, 052401 (2014)

[16] D. Ruzmetov et al. *Phys. Rev B* 79, 153107 (2009)

[17] T.Yamin et al. *Sci. Rep.* Vol 6, 19496 (2016)
